# Supplementary material for: Development of PCR-Multiplex Assays for Identification of the Herpotrichiellaceae Family and Agents Causing Chromoblastomycosis
Source: J Fungi (Basel). 2024 Aug 4;10(8):548. doi: 10.3390/jof10080548 (PMC11355602; doi:10.3390/jof10080548)
Supplement: Supplementary file 1 [file jof-10-00548-s001.zip › Table S1.pdf]

Table S1: Sequences used in the in silico analyses and Genbank code access.

Specie	ITS	LSU
Fonsecaea pedrosoi	NR_130652.1	OK663662.1
Fonsecaea pedrosoi	FJ914990.1	MH878034.1
Fonsecaea pedrosoi	KP132210.1	MH877955.1
Fonsecaea pedrosoi	KP132209.1	MH876658.1
Fonsecaea pedrosoi	KP132208.1	MH875348.1
Fonsecaea pedrosoi	KP132207.1	MH875345.1
Fonsecaea pedrosoi	KY312523	MH875344.1
Fonsecaea pedrosoi	KY312524	MH875343.1
Fonsecaea pedrosoi	KY312525	MH875342.1
Fonsecaea pedrosoi	PP187001	MH875209.1
Fonsecaea pedrosoi	PP187002	MH875208.1
Fonsecaea pedrosoi	PP187003	MH875207.1
Fonsecaea pedrosoi	PP187004	*
Fonsecaea pedrosoi	KY312526	*
Fonsecaea monophora	NR_131280.1	MH878004.1
Fonsecaea monophora	FJ785471.1	*
Fonsecaea monophora	KY432482.1	MH875081.1
Fonsecaea monophora	KY432480.1	FJ358247.1
Fonsecaea monophora	KY432479.1	JQ906794.1
Fonsecaea monophora	KY312534	JQ906792.1
Fonsecaea monophora	KY312538	JN418763.1
Fonsecaea monophora	MF416920	JN418761.1
Fonsecaea monophora	KX078396.1	MH878004.1
Fonsecaea monophora	KX078395.1	MH875202.1
Fonsecaea monophora	KX078394.1	MH875081.1
Fonsecaea monophora	KX078393.1	ON306951.1
Fonsecaea pugnacius	NR_155089.1	*
Fonsecaea pugnacius	KR706554.1	*
Fonsecaea pugnacius	KR706553.1	*
Fonsecaea pugnacius	MH444809.1	*
Fonsecaea nubica	NR_111333.1	MK828350.1
Fonsecaea nubica	KU885982.1	MK828349.1
Fonsecaea nubica	EU938592.1	MK828348.1
Fonsecaea nubica	KY432481.1	MK828347.1
Fonsecaea nubica	KP132199.1	MK828346.1
Fonsecaea nubica	KP132198.1	MK828345.1
Cladophialophora bantiana	GQ258793.1	OL348329.1
Cladophialophora bantiana	KY432483.1	MH877849.1

Cladophialophora bantiana	KP131826.1	MH874588.1
Cladophialophora bantiana	KP131825.1	MH874436.1
Cladophialophora bantiana	KY312527	AB363799.1
Exophiala dermatitidis	NR_121268.1	OR529212.1
Exophiala dermatitidis	KP959250.1	MH878056.1
Exophiala dermatitidis	KP959249.1	MH878059.1
Exophiala dermatitidis	KP959248.1	MH878058.1
Exophiala dermatitidis	KY312530	MH878057.1
Exophiala dermatitidis	MH865405.1	MH876931.1
Exophiala dermatitidis	MH865404.1	MH876930.1
Rhinocladiella similis	NR_166008.1	KU752195.1
Rhinocladiella similis	PP187007	*
Rhinocladiella similis	MW447088.1	*
Rhinocladiella similis	KP132564.1	*
Rhinocladiella similis	KP132562.1	*
Cladophialophora carrionii	EU137294.2	MH873312.1
Cladophialophora carrionii	EU137309.1	MH871463.1
Cladophialophora carrionii	EU137290.1	MH868814.1
Cladophialophora carrionii	EU137304.1	MH868813.1
Cladophialophora carrionii	EU137303.1	*
Cladophialophora carrionii	EU137302.1	*
Phialophora verrucosa	NR_146242.1	MT023621.1
Phialophora verrucosa	KP132498.1	MT023620.1
Phialophora verrucosa	DQ404353.1	*
Rhinocladiella aquaspersa	GU017733.1	MH876010.1
Rhinocladiella aquaspersa	GU053606.1	MH877943.1
Rhinocladiella aquaspersa	MG996793.1	MH872396.1
Rhinocladiella aquaspersa	GU017732.1	MH864573.1
Rhinocladiella aquaspersa	MH445518.1	*
Rhinocladiella aquaspersa	MH444803.1	*
Fonsecaea multimorphosa	NR_111612.1	*
Fonsecaea multimorphosa	EU938595.1	*
Fonsecaea multimorphosa	MH864212.1	*
Exophiala spinifera	NR_111131.1	MH876941.1
Exophiala spinifera	AY156966.2	MH876261.1
Exophiala spinifera	AY156970.1	MH876260.1
Exophiala spinifera	AY156964.1	MH876259.1
Exophiala spinifera	AY156962.1	*
Cladophialophora immunda	NR_111283.1	MH875705.1

Cladophialophora immunda	EU137318.1	MH875704.1
Cladophialophora immunda	FJ385272.2	MH875703.1
Phialophora americana	U31847.1	MH871231.1
Phialophora americana	MT280660.1	MH870708.1
Phialophora americana	MT280659.1	MH859007.1
Cladophialophora boppii	NR_131297.1	MH876019.1
Cladophialophora boppi	KP131827.1	FJ358233.1
Veronaea botryosa	NR_103593.1	OP377938.1
Veronaea botryosa	MG922570.1	MH875937.1
Veronaea botryosa	KP187645.1	MH875936.1
Veronaea botryosa	EU041816.1	MH875352.1
Exophiala lecanii-corni	NR_145351.1	MW807311.1
Exophiala lecanii-corni	MH063099.1	MH867492.1
Exophiala lecanii-corni	MH063098.1	FJ358243.1
Rhinocladiella mackenziei	KP132553.1	OQ804484.1
Rhinocladiella mackenziei	KP132552.1	EU041867.1
Rhinocladiella mackenziei	MF401515.1	EU041866.1
Rhinocladiella mackenziei	EU041809.1	EU041865.1
Exophiala jeanselmei	NR_111129.1	MH873915.1
Exophiala jeanselmei	JN625228.1	FJ358242.1
Exophiala jeanselmei	JX473278.1	MT023628.1
Exophiala oligosperma	NR_111134.1	MH876068.1
Exophiala oligosperma	KY290272.1	MH868049.1
